# Blood–brain borders: a proposal to address limitations of historical blood–brain barrier terminology

**DOI:** 10.1186/s12987-023-00478-5

**Published:** 2024-01-05

**Authors:** Jerome Badaut, Jean-François Ghersi-Egea, Robert G. Thorne, Jan Pieter Konsman

**Affiliations:** 1 Brain Molecular Imaging Lab, UMR 5536, CNRS, RMSB, University of Bordeaux, 146 Rue Léo Saignat, 33076 Bordeaux Cedex, France; 2 Basic Science Department, Loma Linda University School of Medicine, Loma Linda, CA USA; 3 FLUID Team, Lyon Neurosciences Research Center, INSERM U1028, CNRS UMR 5292, Lyon-1 University, Bron, France; 4 Denali Therapeutics, Inc, 161 Oyster Point Blvd., South San Francisco, CA 94080 USA; 5 Department of Pharmaceutics, University of Minnesota, 9-177 Weaver-Densford Hall, 308 Harvard St. SE, Minneapolis, MN 55455 USA; 6 UMR 5164, CNRS, ImmunoConcEpT, University of Bordeaux, 146 Rue Léo Saignat, 33076 Bordeaux Cedex, France

**Keywords:** Blood–brain barrier, Blood–brain interface, Blood–brain border, Transport, Immune system, Neurovascular unit

## Abstract

Many neuroscientists use the term Blood–Brain Barrier (BBB) to emphasize restrictiveness, often equating or reducing the notion of BBB properties to tight junction molecules physically sealing cerebral endothelial cells, rather than pointing out the complexity of this biological interface with respect to its selectivity and variety of exchange between the general blood circulation and the central nervous tissue. Several authors in the field find it unfortunate that the exquisitely dynamic interfaces between blood and brain continue to be viewed primarily as obstructive barriers to transport. Although the term blood–brain interface is an excellent descriptor that does not convey the idea of a barrier, it is important and preferable for the spreading of an idea beyond specialist communities to try to appeal to well-chosen metaphors. Recent evidence reviewed here indicates that blood–brain interfaces are more than selective semi-permeable membranes in that they display many dynamic processes and complex mechanisms for communication. They are thus more like ‘geopolitical borders’. Furthermore, some authors working on blood–brain interface-relevant issues have started to use the word border, for example in border-associated macrophages. Therefore, we suggest adopting the term Blood–Brain Border to better communicate the flexibility of and movement across blood–brain interfaces.

## Introduction

The word “barrier” is derived from the Old French ‘barrière’, denoting a palisade or fortification defending an entrance, and generally refers to a natural or a man-made structure that blocks movement or access. Accordingly, definitions of barrier include words such as ‘barricade’, ‘entrenchment’, and ‘boundary’ (Samuel Johnson Dictionary), phrases such as ‘fence or material obstruction’ (Oxford English Dictionary) or, alternatively, ‘a natural formation or structure that prevents or *hinders* movement or action’ (Merriam-Webster). While most of the above descriptions are more or less limited to a binary interpretation of a barrier as either being intact or broken, the last description, in referencing the concept of *hindrance,* appears to leave room for some selectivity of movement. Interestingly, the authors who first coined the term “hemato-encephalic barrier” (translated from French) importantly considered it “to play the role of a … selective barrier” [1]. Decades later, the advent of electron microscopy favored a view that came to consider the blood–brain barrier in large part as an ‘unbroken belt’ of tight junctions between adjacent cerebral endothelial cells, even though it was appreciated that certain small molecules, such as glucose, must be able to pass through via ‘special transport mechanisms’ [2]. Indeed, common usage in present day biology most often emphasizes the restrictiveness of the blood–brain barrier, equating or reducing the notion of barrier properties to tight junction molecules physically sealing cerebral endothelial cells, thus presenting the brain endothelium as a more or less homogenous ‘palisade’ that fails to capture its true selectivity, variety and complexity.

**Fig. 1:**
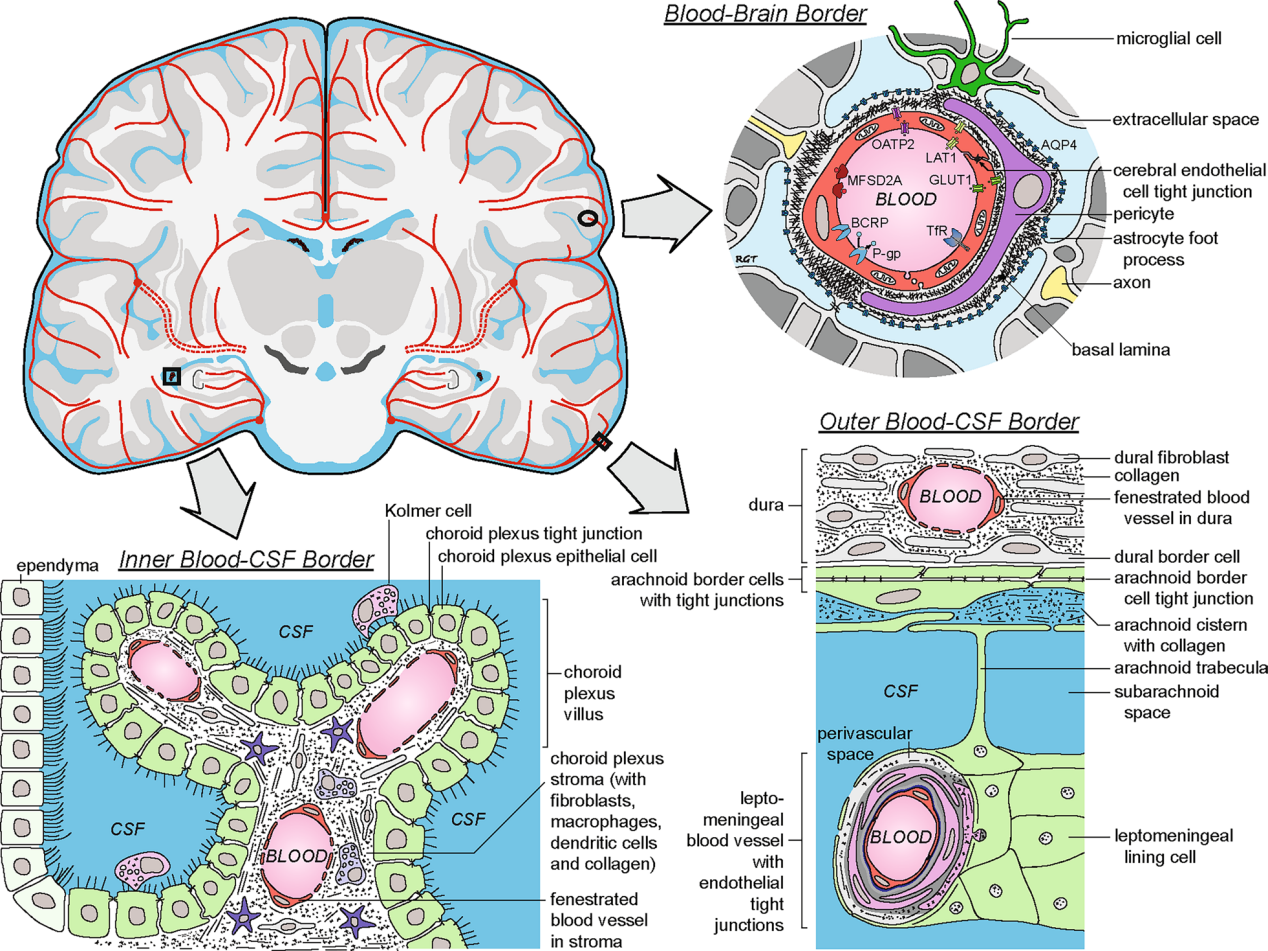
Illustration of key borders between the blood and brain and between the blood and cerebrospinal fluid (CSF). Blood–brain border (neurovascular unit): cerebral endothelial cells in the parenchyma of the brain contain tight junctions and express numerous transporters and receptors that regulate the transfer of substances between the blood and brain (upper right), as shown by the following examples: Efflux transporters (e.g. P-glycoprotein, ABCB1; breast cancer resistance protein, ABCG2) prevent brain entry of many circulating endogenous substances as well as xenobiotics (drugs). The glucose transporter 1 (GLUT1, SLC2A1) and transferrin receptor (TfR, CD71) mediate brain homeostasis of glucose and iron, respectively, through the uptake of circulating glucose and iron-bound transferrin. The major facilitator superfamily domain containing 2a (MFSD2A) is a fatty acid transporter that is specifically expressed in CNS endothelial cells; MFSD2A also serves an important role in inhibiting clathrin-independent caveolae-mediated transcytosis [37, 38]). Outer blood-CSF border (meninges): outer arachnoid epithelial border cells contain tight junctions and express numerous transporters and receptors (not shown) that potentially assist in the regulated transfer of substances between the blood and extraventricular CSF (bottom right). Inner blood-CSF border (choroid plexus): choroid plexus epithelial cells contain tight junctions and express numerous channels, transporters, receptors and enzymes (not shown) that regulate the composition of CSF (bottom left). As examples, a finely tuned interplay between apically and basolaterally located inorganic anion transporters and channels is responsible for CSF production [39]. Transporters of the ABCC, SLC0, SLC21 families control CSF concentration of potentially deleterious endo and xenobiotics [4], helped by efficient enzymatic detoxification mechanisms [40]. The choroidal transport or secretion of growth factors, hormones and proteins into the brain participates in processes essential for brain development and homeostatic balance [33, 41]. Schematics based in part on several sources [12, 42–44]

Authors active in the blood–brain barrier field, including Bill Banks, Ian Galea, Hugh Perry, and Aravinthan Varatharaj have expressed reservations [5–7, 122] that these exquisitely dynamic interfaces between blood and brain, capable of important adaptations and plasticity over the life span, continue to be considered by many neuroscientists and others in the wider scientific community primarily with respect to the blocking aspects of barrier properties. Another impediment to the usefulness of the blood–brain barrier concept in mainstream usage is that it overlooks the fact that exchanges between the blood and the brain occur not only across the extensive surface area of the brain’s blood vessels, but also at several other important anatomical sites (Fig. 1). It is also increasingly apparent that there is significant variation in brain endothelial cell properties at different sites along the brain’s blood vessels, e.g. single cell transcriptomics studies have shown dramatic variation in cellular protein expression patterns at different points along the cerebrovascular tree, a notion referred to as brain endothelial arteriovenous zonation [3]. Accordingly, the numerous contact points between the blood and the CNS are therefore quite complex and varied; far from being a simple, single type of interface, they actually include a number of unique, heterogeneous interfaces that allow for many different types of communication and exchange with the rest of the body. Several excellent reviews have recently pointed out the limitations of standard blood–brain barrier terminology and have proposed the adoption of a more neutral and descriptive concept, e.g. blood–brain interfaces [4–7]. We would like to suggest a new concept that uses the geopolitical metaphor of ‘border’ to better capture properties of these important biological interfaces and more easily allow other scientific domains to grasp their essence.

Taking into account the selective, dynamic and adaptive aspects of the blood–brain interfaces together with inspiration from the domain of political philosophy [8], we believe that the notion of a ‘border’, rather than that of a ‘barrier’, better captures the properties of these interfaces. We will review the importance of passive diffusion, transport systems, perivascular cell dynamics and immune cell traffic between blood and brain and argue in favor of the concept of a blood–brain border to replace that of a blood–brain barrier.

## Restricting properties of the brain barriers

*“The wall is the rule … that unifies all of the various divided subgroups into a higher unity while at the same time retaining the partition fences and organizing them into bricks.”* [8], p. 65).

The discovery and functional characterization of the tight junctions have been a major advance in the understanding of blood–brain interface ‘fence-wall’ functions, but at the same time acknowledgment of brain permeation to endogenous molecules was thus mainly reduced to the ‘exception’ of specific transport systems for glucose and essential amino acids. Indeed, when the barrier properties of the blood–brain interface(s) preventing intravenously-injected molecules to enter the brain parenchyma via paracellular diffusion were observed with electron microscopy to coincide with that of so-called tight junctions between adjacent cerebral endothelial cells [2, 9, 10], choroid plexus epithelial cells [11], and outer arachnoid epithelial cells [12], the idea of selectivity was put in the background, at least temporarily. Indeed, during the second half of the twentieth century, work on the blood–brain barrier in relation to tight junctions [10, 13, 14] somewhat downplayed barrier selectivity concepts that had been elaborated half a century earlier [1]. The notion of selectivity was then gradually reintroduced when several molecules, including hormones, were shown to be transported across the blood–brain interface (see for early reviews [15–17]).

Against this historical background, it is not surprising that brain barriers have initially been viewed only as a physical hindrance to therapeutic drug diffusion into the brain. For a long time, the permeability of the blood–brain barrier to a pharmacological compound was considered to dependent simply on the size of the molecule and its ability to cross the cell membranes. Hence, many past efforts from pharmaceutical companies to develop CNS drugs were targeted toward the development of small molecules and structural modifications were oriented toward an increase in the lipophilicity of the compound. Once efflux transport proteins were discovered at the blood–brain barrier, beginning with p-glycoprotein [18, 19], the notion of an absolute barrier preventing even lipophilic drugs from entering the brain was further strengthened. The more recent development of large molecules with therapeutic potential, e.g. peptides, antibodies, enzymes, oligonucleotides and other biologics, has forced a reevaluation of the mechanisms allowing macromolecules to bypass the ‘barrier’ and, in the process, opened up new avenues for drug delivery into the brain [20]. Targeting brain endothelial cell-enriched receptors such as the transferrin receptor with antibodies has been pursued for decades [21–24]. More recently, engineered transport vehicles harnessing transferrin receptor-mediated transcystosis across brain endothelial cells [25, 26] have shown promise and are now under evaluation in the clinic for the treatment of mucopolysaccharidosis type II, a rare, inherited genetic disorder with frequently severe neurologic involvement (clinicaltrials.gov, NCT 04251026). All of this interest in CNS drug delivery has sparked a vast amount of new research towards a better understanding of blood–brain exchanges and their plasticity.

Despite widespread appreciation among specialists that many substances, including certain large hydrophilic molecules, can in fact be transported across the cerebral endothelium, many reviews intended at non-specialists have continued to focus predominantly on tight junction molecules between brain endothelial or epithelial cells [27, 28]. After relating the accumulation of circulating dyes at the levels of tight junction molecules, such reviews would typically state that the “tightness” of these junctions “is best reflected by their [high] electrical resistance” [27]. This measure of transendothelial or transepithelial electrical resistance (TEER) has today become a widely accepted quantitative technique to evaluate cell culture models of biological barriers, including the blood–brain barrier [29].

The barrier between the blood and the ventricular cerebrospinal fluid (CSF), located at the epithelium of the choroid plexuses, is often qualified as more ‘leaky’ than the blood–brain barrier. Among the reasons put forward are the ability of the epithelium to secrete fluid, along with a reportedly low TEER (~ 150 Ω·cm^2^ in bullfrog; [30]) as compared to what has been measured across endothelial cells of pial blood vessels (~ 1500–2000 Ω·cm^2^; [31, 32]). However, these particularities are not the result of mere loose tight junctions. The word ‘leakiness’ does not do justice to the multiple channels, carriers, and receptor-mediated transcytosis systems that are active at the blood-CSF barrier. Rather, it is likely that multiple highly regulated and energy-demanding mechanisms (necessitating a high mitochondrial activity), along with higher rates of vesicular trafficking, explain the fluid secretion and particular border functions of the choroidal epithelium. While choroidal epithelial cells display efficient tight junctions that hinder water-soluble compounds from reaching the CSF by a paracellular path, they also allow regulated transcellular transport and secretion of a variety of nutrients, hormones, cytokines, and other plasma components through mechanisms that often differ from those at the blood–brain barrier proper [33]. For example, it has long been appreciated that small amounts of numerous circulating plasma proteins may efficiently access the CSF, with CSF:serum ratios that drop with protein molecular weight [34], and that selected epithelial cells are dedicated to the blood-CSF transfer of plasma proteins [35, 36].

## Flexibility of the blood–brain interfaces

“*The first port wall is the transport wall, which regulates the circulation ….*” [8], p. 81)

“*The Romans were the closest to achieving this kind of port at Hadrian’s Wall. The primary function of Hadrian’s Wall was not to defend against barbarian invasion, but to regulate the ports of entry into the empire and collect taxes from those who wanted to pass across its numerous gates built at each milecastle.*” [8], p. 86)

The importance of the selective trading/exchange at the borders between the central nervous system tissues and the blood stream is well illustrated by the idea of multiple gates allowing for exchanges of different nature between blood and brain, and by the differentially-expressed transport proteins at these borders contributing to selective influx and efflux of substrates in and out of the central nervous system (Fig. 1).

In addition, the dynamics and plasticity of blood–brain interfaces are lifelong phenomena that are observed as early as the brain is developing. For example, it is well established that many amino acids and metabolically-active compounds are transported into the developing brain at much higher rates than into the adult brain [45]. This observation is consistent with the greater metabolic demand of the developing brain and is due to regulated transport, even though some authors have incorrectly suggested that greater uptake may be due to a leaky and/or immature blood–brain interface [46]. The latter idea seems to be based, in part, on a widespread mistaken belief regarding ‘immaturity’ of blood–brain barrier properties [47]. With respect to an elevated need for amino acids during brain development, it is not surprising that recent work has shown higher expression of many specific transporters at blood–brain interfaces of the cerebral endothelium and choroid plexuses in neonatal animals as compared to adults [48, 49]. Many members of the solute carrier (SLC) superfamily transporters are expressed at higher levels in the developing brain compared to the adult, thus accounting for the earlier observations of higher entry of specific amino acids into the developing brain [50]. The need for higher expression of some transporters in relation to metabolic demand should be considered against the background that nascent blood vessels express tight junction molecules that impair paracellular diffusion at the moment they initially grow into neural tissue, i.e. ‘barrier’ properties arise with the onset of vasculogenesis [51–54]. One way to interpret this phenomenon is that the blood–brain interface adapts transport according to the need to ‘import’ certain substrates required for the adequate development and functioning of neural tissue.

Today, it is appreciated that many hormones and cytokines are selectively transported across the blood–brain interface made up by tight-junction-expressing endothelial cells [55, 56] and that pro-inflammatory cytokines can signal across the blood–brain interfaces during systemic inflammation [57]. It is also acknowledged that developmental stage-specific secretion or transport of trophic factors occurs at the blood-CSF border according to neural precursor needs [58], and that plasma-to-brain transport of circulating proteins may change with ageing [59, 60].

**Fig. 2:**
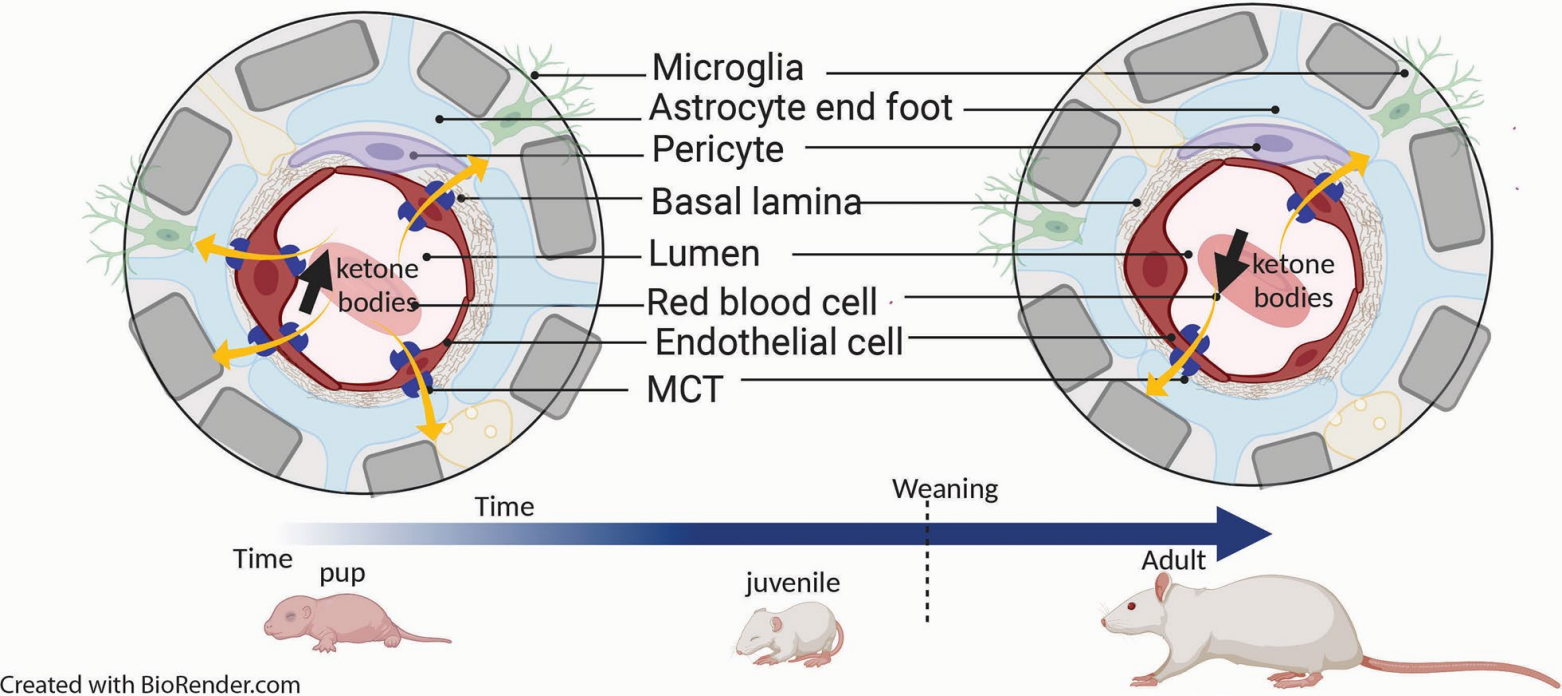
Illustration of the physiological adaptation of a number of transporters on brain endothelial cells with the transition from the use of the ketone bodies during lactation to that of mostly glucose after weaning. The endothelial cells adapt the number of monocarboxylate transporters (MCT in Blue) in function of the need of the energy substrate, with higher number of MCT during lactation to facilitate the ketone bodies to fuel the brain than in the adulthood with the use of glucose from the general blood circulation. It is similar to a country facilitating product importation in function of its needs (Fig. 3)

**Fig. 3:**
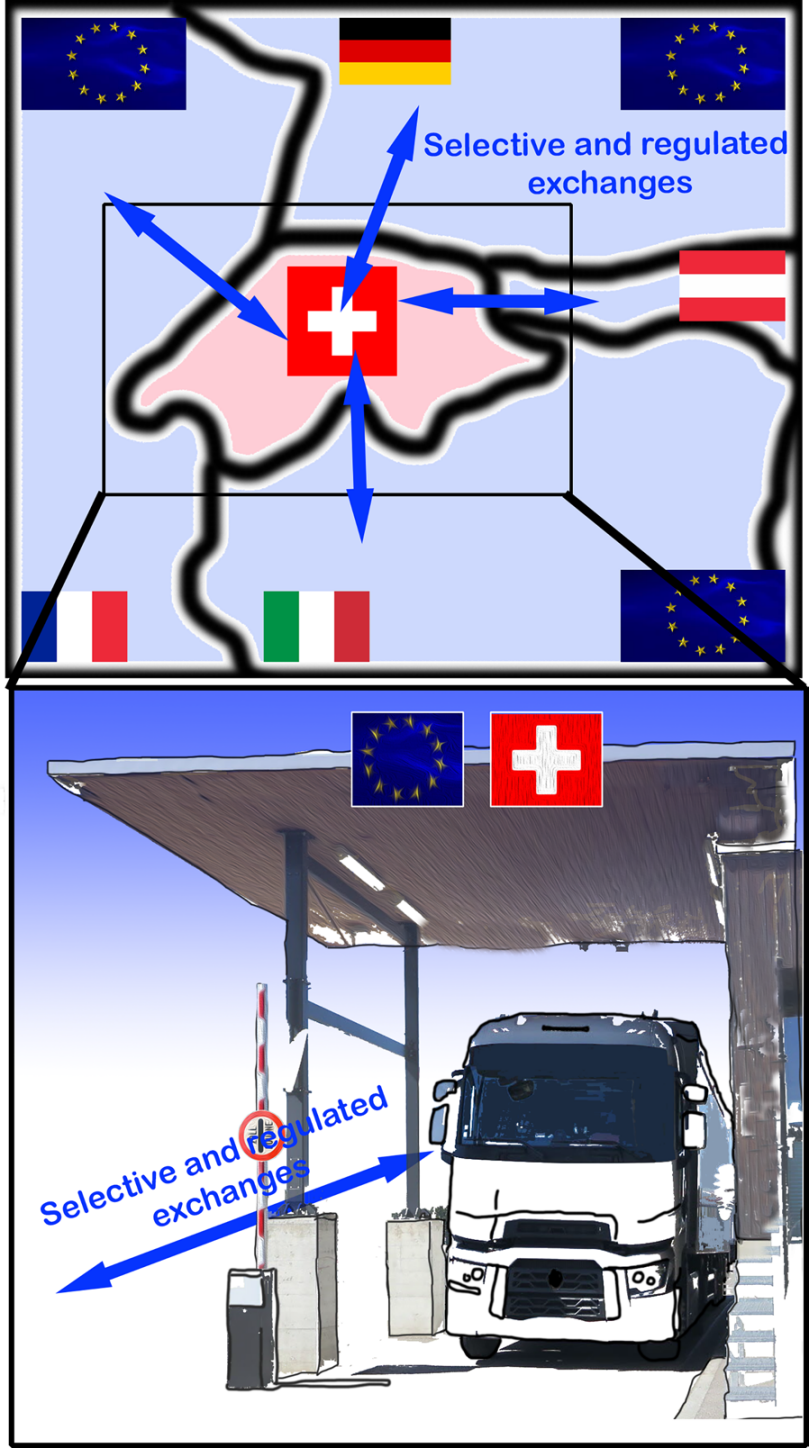
Schematic Illustration of the trade between Switzerland and E.U. with changes and adaptations over time depending on the needs of each partner. The supply exchange at the custom border is controlled and selective with potential taxes fixed (before) by the partners

In addition to amino acids, hormones and cytokines, endothelial cells at the blood–brain interface can also regulate transport of energy substrates such as lactate and other monocarboxylates into and out of the brain by controlling expression of the monocarboxylate transporter 1 (MCT1), e.g. increasing MCT1 expression when the organism is on a ketogenic diet in order to facilitate extraction of plasma ketone bodies by the brain [61, 62]. Ketone bodies can be used as an energy substrate by the brain when a diet not containing any glucose is consumed such as during lactation. At the time of weaning, expression of MCT on endothelial cells decreases in parallel with the shift to glucose use (Fig. 2). Regulation of transporters has also been demonstrated in the study of the effects of brain ischemia. This condition induces expression of the transcription factor hypoxia-inducible factor-1 (HIF-1) in brain endothelial cells, which, in turn, upregulates glucose transporter 1 (GLUT1) expression and thus facilitates the entry of glucose into the brain [63]. Hence, work spanning the last decades has greatly expanded our knowledge of regulated substrate transport at the different interfaces between the blood and neural tissue. This type of regulation of substrate movement is in many respects similar to what countries do with regards to import and export rules (e.g. Switzerland and E.U. countries) in order to sustain their economic activity despite the existence of borders with other countries. Trading and taxes on importation and exportation change over time for countries depending on their specific needs (Fig. 3).

In brain endothelial cells, selective transporters facilitating the entrance of some substrates co-exist with ‘gatekeepers’ that limit the entry of molecules that are toxic to neural tissue. Many of these ‘gatekeepers’ are part of the ATP-binding cassette (ABC) transporters, e.g. p-glycoprotein (P-gp; ABCB1). P-gp contributes to limit the entry of toxins into the CNS from the bloodstream as well as to the brain-to-blood clearance of beta-amyloid. Importantly, the level of expression of this transporter decreases with ageing [64] and after a traumatic brain injury [65]. Decreased P-gp expression leads to increased transport of certain substrates across the border into the brain while efflux of beta-amyloid out of the brain decreases, with resulting detrimental effects to the CNS microenvironment [65, 66]. This situation further illustrates how key properties of the blood–brain interface may change over time. In contrast, increased brain endothelial P-gp expression has been demonstrated in a model of peripheral inflammatory pain, and this mechanism may participate to the reduced efficacy of morphinic analgesic drugs by reducing their delivery to the central nervous system [67]. Interestingly, the level of P-gp expression in endothelial cells appears to be be under the control of Caveolin-1, a key scaffolding/trafficking protein that is composed of plasmalemmal cholesterol and sphingolipid-rich raft subdomains that take part in endocytosis of caveolae, which, in turn, control transcellular permeability of this blood–brain interface [37, 68]. The clathrin-coated caveolar membranes can contain receptors for transferrin, insulin, albumin, ceruloplasmin, RAGE, LDL, LRP1, HDL, interleukin- 1, and vesicle-associated membrane protein-2 [69], which strengthens the idea that transcytosis across the endothelial blood–brain interface occurs only under the control of specific receptors, a situation not unlike what occurs at the borders between countries. Although studies of transcytosis across the blood-CSF interfaces are more limited, it has emerged that transcytosis pathways are typically receptor-mediated and highly regulated also at these sites [70]. These, as well as the efflux processes clearing the CSF from deleterious metabolites, call upon carriers and receptors that differ from those present at the cerebral endothelium (Fig. 1), illustrating the functional complementarity of the different ‘check points’ along blood–brain borders.

With these examples, it becomes clear that the blood–brain interfaces are genuinely complex molecular machineries. And just as country borders evolve over time, blood–brain borders also change throughout life depending on both physiological and pathophysiological conditions.

## Surveillance at the blood–brain borders

“*Like all border limits, the territorial wall does not define a permanent or fixed place, but a defensive buffer zone or supply line that retraces the military march.*” [8], p. 79)

The CNS has long been considered to be an immune-privileged site within the body. However, this idea has often been translated to the brain being immune-deprived as many prototypical immune cells, such as T-cells and dendritic cells, were only sporadically found. Moreover, in the mind of many scientists and clinicians the idea of a blood–brain barrier implied that no immune cells could enter the CNS [71, 72]. In this part, the emergence of the idea that immune cells enter the CNS even in the absence of blood–brain barrier ‘breakdown’ will be developed [73, 74]. Here, the case will be made that it makes heuristically more sense to try and articulate the military-inspired metaphors for immune cells [73, 74] with a border, rather than with a barrier metaphor for the blood–brain interface.

Activated T-lymphocytes, regardless of their antigen specificity, have long been known to cross the blood–brain interfaces and enter the brain [33, 75, 76]. They have been proposed to take part in patrolling and surveillance of the CNS [77–80], even though they do not normally leave the interconnected perivascular/CSF spaces [81]. Interestingly, astrocytes have been proposed to form borders with their endfeet along all perivascular spaces and meninges that separate immune-cells from neuronal tissue and to restrict CNS parenchymal access of leukocytes and hence inflammatory responses [82]. For these T-cells to exert action, they need to be presented with antigen. Although dendritic cells, as professional antigen-presenting cells are scarce in the CNS, some resident brain cells have the capacity to present antigen. Among these, perivascular and meningeal myeloid cells, together with the choroidal macrophages, are part of fully functional macrophages situated at the different blood–brain interfaces [83–88], in contrast to brain parenchymal microglia that require several hours of activation time before they display macrophages characteristics, such as phagocytosis. Interestingly, in recent publications, perivascular macrophages have also been referred to as “border-associated macrophages” (BAMs) [89–91]. Given that perivascular, meningeal and choroid plexus macrophages have many cell markers in common [88], it would make sense to refer to all of these cells as “border-associated macrophages”, the idea of border being introduced in order to illustrate patrolling of cells along the endothelial or epithelial layer which can be viewed as gates to the brain.

Brain perivascular macrophages are thought to constitute a ‘line of defense’ through phagocytosis of particles present in the perivascular space and to play a role in immune surveillance [85, 92–94]. Both phagocytosis and immune surveillance may be facilitated by the continuous retraction and protraction of macrophage processes along blood vessels [95], like ‘patrols guards’ present at the ‘border gate’. In addition to brain perivascular macrophages, it will also be interesting to elucidate the function of meningeal macrophages and their relationship to the lymphatic vessels that have been described within the dura mater [96, 97]. Finally, the particular immune cell content and distribution within the choroidal tissue deserve mention. Dendritic cells and macrophages are located on the blood side within the choroid plexus stroma, while macrophages (Kolmer cells) can be found attached to the epithelium on the CSF side (Fig. 1). Each of these cell types can also be interpreted as and likened to patrol guards on different sides of a border with communication occurring between both sides. Indeed, this organization might be related to the controlled traffic of both acquired and innate immune cells occurring at this interface both during neuroimmune surveillance and in pathological situations [33, 98–100].

In crisis situations, brain meningeal and perivascular macrophages have been shown to be protective in a model of bacterial meningitis and this may be related to their role in promoting the recruitment of circulating leukocytes [101]. Depletion of meningeal and brain perivascular macrophages also decreases the clearance of extracellular fibrinogen in the meninges after mild traumatic brain injury indicating that these cells play a role in wound healing [102]. However, the role of meningeal and brain perivascular macrophages in granulocyte recruitment and in the increase of the permeability of pial and cortical blood vessels does contribute to neurological dysfunction during the acute phase of ischemia/reperfusion [89].

These findings indicate that meningeal, choroidal and brain perivascular macrophages are important in response to acute infection and brain injury [94]. The roles that these brain macrophages play, in addition to their strategic positioning in what can be considered to be defensive buffer zones along blood–brain interfaces, fully justifies the name border-associated macrophages and further encourages us to consider these interfaces as biological borders. It is, however, important to keep in mind that just like some changes in endothelial cell properties can be beneficial to functioning of the neurovascular unit whereas other modifications can contribute to the pathophysiology of CNS diseases, some changes in brain border macrophages may also turn out to be beneficial for cerebral function.

## Why ‘border’ would be a better metaphor than ‘barrier’ to convey the complexity of the blood–brain interface

Experimental scientist often tend to think that words have little importance and that all matters are observations and measures. It is, therefore, key to point out that metaphors are present in science, including in immunology and neuroscience, and often serve to communicate ideas not only between different fields and disciplines of science, but also towards a lay audience. However, a metaphor used to communicate scientific ideas may be more or less heuristically useful, valid or relevant and these questions have given rise to a lot of debate [103–105]. One common position in such debates is the recommendation to stop using a metaphor because “outdated metaphors may limit scientific inquiry and contribute to public misunderstanding” [106]. With this in mind, the recent proposal by some authors of the descriptive blood–brain interface rather than the metaphoric blood–brain barrier [5, 6] is a welcome step forward.

It is however important to consider “the explanatory function of metaphor” [107], p. 157) in that a “metaphor has the power to further the students’ understanding of the scientific concept at hand” [108], p. 89). More broadly speaking, metaphors can help to motivate and mobilize certain resources as well as to influence the direction of research programs [109]. For example, and as alluded to above, military-inspired metaphors are widely used for immune cells. Numerous metaphors are being used to try and characterize our understanding of brain structure and function [110, 111]. Interestingly, the metaphor of the brain as a fortress or castle that has to be opened is often used [111]. Although, the sense in which fortress is used for the brain is mostly that of a black box containing secrets that need to be rendered visible, it is nevertheless interesting to relate this to the image of the brain as a castle, which has been employed to present the blood–brain interface as the “two-walled castle moat surrounding the CNS castle” with endothelial cells as a first wall and astrocyte endfeet as a second wall [112, 113]. However, the idea of a two-walled castle moat still seems close to that of a barrier with the addition of the perivascular space as a place for cell circulation behind the first wall represented by endothelial cells. It misses the selectivity aspect of the initial definition given by Stern & Gautier of the term hemato-encephalic barrier. In contrast, the metaphor of a border seems to do justice to this selectivity aspect of the blood–brain interface and is therefore worthwhile exploring.

As pointed out above, several recent publications have referred to meningeal and choroid plexus macrophages as border-associated macrophages [89–91]. Furthermore, the term border has repeatedly been used to describe the choroid plexus epithelium [114, 115] as well as the outer arachnoid epithelium (arachnoid border cells [12]. The idea to apply the metaphor of border to the blood–brain interfaces in addition to that of the blood-CSF interface is corroborated by the fact that several authors have already employed the word border to refer to the blood–brain barrier proper. For example, when considering strategies for delivery of molecules of therapeutic interest to brain tissue, a science writer relates how researchers “hope to find out how this border control manages to pick and choose which particles it lets through, in the hope that it will help drug designers to target the brain more effectively” [116]. Several review papers on immune cell extravasation in relation to the blood–brain interface refer to it as the “endothelial [cell] border” [117, 118] or “border structures” [119].

Any given border between countries is known to evolve over time, as demonstrated both by historical and recent examples, with changes in the ‘filtering capacities’ of a border in reaction to conflicts and trading agreements. A brain endothelial cell can modulate the level of expression of its transporters or transcytosis properties in response to changes in physiological and pathophysiological status [120, 121]. These adaptations show the flexibility and the dynamic complexity of blood–brain interfaces.

We believe it is also important to highlight that the word barrier is prone to generate misconceptions regarding brain endothelial cell pathophysiology. For example, oft-used terms such as barrier disruption, barrier rupture, barrier breakdown and barrier opening clearly do a disservice to the complex, multifaceted processes underlying dynamic changes in permeability at the blood–brain and blood-CSF interfaces. Indeed, it is well established that changes in permeability at the blood–brain interfaces may occur independently of the physical loss of tight junctions, and can be mediated by physiological responses regulating tight junction permeability as well as transcytosis, e.g. through increased formation of caveolae [121–123]. In this regard, we are convinced that the term **border** does more justice to the flexibility observed in the (patho)physiology of the blood–brain interface, while avoiding the negative connotation of disruption or rupture. It is also quite important to realize that changes in endothelial cell properties can be beneficial to the neurovascular unit and its functioning.

Finally, the political philosophy reference we referred to throughout this article considers that the term border covers historical examples of wall-based enclosures [8]. However, the author aims “to reveal the mutable and arbitrary nature of … dominant border regimes” and “to interpret … them according to the very thing they are supposed to control: movement” [8], p. 5). It is our goal to try and transpose this idea to borders controlling movement of biological entities.

We hope that adoption of the border instead of the barrier as a metaphor to describe blood–brain and blood-CSF interfaces might stimulate new drug approaches to modulate the properties of CNS endothelial and epithelial layers or take advantage of endogenous systems rather than finding ways to’break the barrier’ to facilitate drug delivery to the CNS. Adoption of the border metaphor will acknowledge all the recent work describing the dynamic and plastic nature of the blood–brain interfaces. The border metaphor may also be easily extended to a vast range of other sites of contact between different CNS compartments, e.g. the ependyma within the ventricles, the pia-glia limitans at the brain surface, and the leptomeningeal cells forming the interface between the CSF and the perivascular fluid of subarachnoid blood vessels. For example, while the concept of a ‘barrier’ between the brain interstitial fluid and CSF strikes many as inaccurate, there can be no debate that the pia-glia limitans and ependymal *borders* between brain and CSF regulate transport and exchange in complex ways that are only just beginning to be better understood [43]. Taken together, all of the different CNS borders can be appreciated as unique and varied places of communication and exchange with the rest of the body.

## Conclusion

“*Borders … are neither statist, nor fixed, nor designed to stop human movement. Borders are not permeable membranes that people pass through. They are themselves mobile processes designed to redirect, recirculate, and bifurcate social motion-not stop it*.” [8], p. 221)

In summary, we have reviewed classic and more recent literature to make the point that the blood–brain interfaces should not be considered a simple physical barrier, but rather as a selective barrier as initially proposed [1]. Although the term blood–brain interface is an excellent descriptor that does not convey the idea of a barrier, it is important and preferable for the spreading of an idea beyond specialist communities to try to appeal to well-chosen metaphors. Recent knowledge shows that blood–brain interfaces are more than “permeable membranes” but also that “they are mobile processes” and are thus more like “borders” in the sense of the recent “theory of the border” elaborated by Thomas Nail in political philosophy [8], p. 221). Furthermore, some authors working on blood–brain interface-relevant questions have started to use the word border, for example in border-associated macrophages. Therefore, we believe that it is time to adopt the concept of multiple Blood–Brain Border (BBB) sites to reflect all the recent work describing the flexibility of and movement across membranes located between the CNS and the blood. To accompany this proposal to move from ‘barrier’ to ‘border’, conference symposia and surveys will be organized.

## Data Availability

Not applicable.
